# *Pueraria lobata* root polysaccharide alleviates glucose and lipid metabolic dysfunction in diabetic db/db mice

**DOI:** 10.1080/13880209.2021.1898648

**Published:** 2021-04-01

**Authors:** Dan Luo, Xiaokang Dong, Jie Huang, Chengcheng Huang, Guowei Fang, Yanqin Huang

**Affiliations:** a Department of Endocrinology, Affiliated Hospital of Shandong University of Traditional Chinese Medicine, Jinan, Shandong, PR China; b Department of Cardiovascular, Affiliated Hospital of Shandong University of Traditional Chinese Medicine, Jinan, Shandong, PR China; c School of Health, Shandong University of Traditional Chinese Medicine, Jinan, Shandong, PR China; d Clinical Education Management Division, Affiliated Hospital of Shandong University of Traditional Chinese Medicine, Jinan, Shandong, PR China

**Keywords:** Hyperglycaemic, insulin resistance, PI3K/AKT, kudzu root, hyperlipidaemia

## Abstract

**Context:**

*Pueraria lobata* (Willd.) Ohwi (Fabaceae) root extract can lower blood glucose levels; however, whether *Pueraria lobata* root polysaccharide (PLP) possesses these effects is still unknown.

**Objective:**

This study evaluates the therapeutic effect of PLP on diabetic metabolic syndrome.

**Materials and methods:**

The db/m mice were assigned to normal control group (NC), db/db mice were divided into four groups randomly (n = 8). The db/db mice received rosiglitazone (10 mg/kg BW) or PLP (100 or 200 mg/kg BW) via oral gavage for 6 weeks. Afterward, blood glucose, insulin, and glycogen content were assayed, and insulin tolerance test (ITT), oral glucose tolerance test (OGTT) were performed. Glucose and lipid metabolism-related parameters and gene expression levels were assayed by ELISA and RT-PCR, respectively.

**Results:**

After treatment with HPLP, the values of body weight, epididymal fat, subcutaneous fat, fasting blood glucose, insulin, and HOMA-IR decreased to 45.89 ± 1.66 g, 1.65 ± 0.14 g, 1.97 ± 0.16 g, 14.84 ± 1.52 mM, 9.35 ± 0.98 mU/L, and 5.56 ± 1.26, respectively; the levels of TG, TC, LDL-C, and FFA decreased to 1.67 ± 0.11 mmol/L, 6.23 ± 0.76 mmol/L, 1.29 ± 0.07 mmol/L, and 1.71 ± 0.16 mmol/L, respectively. HPLP down-regulated PEPCK, G6PC, FOXO1, SREBP-1, and ACC mRNA expression (*p* < 0.01), and up-regulated GS, Akt2, PI3K, GLUT2, PPARα, and LDLR mRNA expression in the liver (*p* < 0.01).

**Discussion and conclusion:**

PLP exerts antidiabetic effects via activating the PI3K/AKT signalling pathway, thus improving insulin resistance, glucose, and lipid metabolism in db/db mice. Thus, PLP may be considered as a potential antidiabetic agent in clinical therapy.

## Introduction

Type 2 diabetes mellitus is a chronic metabolic dysfunction disease, which caused an abnormal carbohydrate, fat, and protein metabolism, which is characterized by fasting and postprandial hyperglycaemia (Ceriello 2005). The persistence of hyperglycaemia is the main risk factor for many chronic diseases, including kidney failure, blindness, cardiovascular disease, and diabetic liver disease (Duckworth 2001; Reidy et al. 2014; Hsiang et al. 2015). At present, the treatment of type 2 diabetes is still a challenging study, because presently accessible hypoglycaemic agents are mostly synthetic drugs, such as thiazolidinediones, acarbose, metformin, rosiglitazone, and insulin. The use of those drugs is limited due to drug resistance and some side effects (Hung et al. 2012). Nowadays, some reports indicate that agents derived from natural products exert antidiabetic effects with lower toxicity and side effects (Patle et al. 2020). Hence, healthier and safer therapies, such as therapeutic use of plant-derived natural products, are urgently demanded.

Natural plant polysaccharides have been shown to possess potential antidiabetic effects. For example, *Rosa roxburghii* Tratt (Rosaceae) (Ci Li) fruit polysaccharide alleviates hyperlipidaemia and hyperglycaemia in type 2 diabetic mice (Wang et al. 2020). Mulberry fruit polysaccharide exerts antidiabetic effects via modulating gut microbiota (Chen et al. 2018). Fructus Mori polysaccharide possesses antidiabetic and antioxidant effects in STZ-induced diabetic mice (Chen et al. 2017). Insulin resistance plays an important role in the pathogenesis of type 2 diabetes and is induced by damaged insulin signalling pathways (Abdin et al. 2010). Previous reports have indicated that some important insulin signalling pathway, including protein kinase B (Akt), glucose transporter 2 (GLUT2), and phosphatidylinositol-3-kinase (PI3K), have been considered as potential targets to regulate insulin resistance and glucose metabolism in type 2 diabetes (Zhu et al. 2013; Hu et al. 2014). *Ophiopogon japonicus* (Thunb.) Ker Gawl (Asparagaceae) polysaccharide has antidiabetic effect via regulating the Glut-4/PI3K/Akt signalling pathways in diabetic mice (Wang et al. 2012).

*Pueraria lobata* (Willd.) Ohwi (Fabaceae) root, also known as kudzu root, is widely distributed in China, Japan, and Korea; it has been used as a medicine, fodder, and a food source for thousands of years. Kudzu root also has been utilized widely as a traditional Chinese Medicine for the treatment of cardiovascular diseases, diabetes, diarrhoea, and fever (Wong et al. 2011). Polysaccharides and flavonoids are the major bioactive constituents in kudzu root, and some studies about the antidiabetic potential of puerarin have been reported (She et al. 2014; Zhang et al. 2015). However, there is little research about the potential anti-diabetes efficacy of *Pueraria lobata* root polysaccharide (PLP).

Thus, the objectives of the present research were to explore the antidiabetic potential and underlying mechanism of PLP in diabetic db/db mice. And our findings will provide a novel therapy in the prevention and treatment of type 2 diabetes mellitus.

## Materials and methods

### Plant materials and chemicals

The dried roots of kudzu root were purchased from Haozhou medicinal material market (Anhui, China) and harvested in October 2019, and authenticated by Jie Huang of the Shandong University of Traditional Chinese Medicine (voucher specimen 20191101). The crude polysaccharide (PLP) used in the present study was extracted from kudzu root according to the previous report (Dong et al. 2020). All other chemical reagents used in the present study were of analytical grade.

### Animal experiments

Male C57BL/KsJ-db/db (db/db) mice and their nondiabetic lean littermates (db/m) (6–8 weeks of age) were purchased from the Laboratory Animal Centre of Shandong Province and were housed under the regulated environmental conditions (12 h dark/light cycle; relative humidity: 45–50%; room temperature: 25 ± 1 °C). All animals were allowed free access to water and diet *ad libitum*. Animal experiments were performed with strict guidelines of Animal Care and Use of Affiliated Hospital of Shandong University of Traditional Chinese Medicine.

### Animal experimental protocol

To better ensure adequately minimalizing the number of mice, the sample size was selected according to previous studies (Khalilpourfarshbafi et al. 2019; Wang et al. 2020). Following acclimatization for 7 days, db/m mice (n = 8) were assigned to the normal control group (NC), db/db mice were divided into four groups randomly (eight mice of each group) as follows: diabetes control group (DC), low-dose PLP group (LPLP, 50 mg/kg body weight), high-dose PLP group (HPLP, 100 mg/kg body weight), and rosiglitazone group (RSG). The RSG mice received RSG (10 mg/kg body weight) daily via oral gavage for six weeks. The LPLP and HPLP mice received PLP at a dose of 50 mg/kg body weight and 100 mg/kg body weight daily via oral gavage for six weeks, respectively. Meanwhile, the DC mice received an equal volume of saline daily via oral gavage for six weeks. The dose of PLP was based on previous literature (Jiang et al. 2013). The PLP or RSG was dissolved in saline. All mice were fed the same standard diet. Bodyweight and food intake were recorded during the experimental period.

### Sample collection

At the end of the experiment, all animals were fasted overnight and sacrificed. Then, the liver tissues, epididymal fat, and subcutaneous fat were quickly collected and weighted. The liver tissues were stored at −80 °C for further analysis. Blood samples were collected and immediately centrifuged at 3000 *g* for 15 min to collect serum for the measurement of biochemical parameters.

Oral glucose tolerance test (OGTT) and intraperitoneal insulin tolerance test (IPTT) were performed. After six weeks of treatment, all animals were fasted overnight. For OGTT, mice were orally administered glucose (2 g/kg body weight). For IPTT, mice were intraperitoneally injected with insulin (2 units/kg body weight). Blood glucose levels were assayed from the tail vein at 0 min (before insulin or glucose administration), 30, 60, 90, and 120 min (after the insulin or glucose loading).

### Determination of fasting blood glucose and fasting insulin concentration

After the mice fasted overnight on the last day, blood was collected from the tail vein. Fasting blood glucose level was measured using commercial kits obtained from Nanjing Jiancheng Bioengineering Institute (Jiangsu, China) following the supplier’s instructions. Fasting insulin concentration was measured using an insulin enzyme-linked immunosorbent assay (ELISA) kit (Thermo Scientific) according to the manufacturer’s protocol. Homeostasis Model of Insulin Resistance (HOMA-IR) was assayed by the following formula: HOMA-IR = (Fasting blood glucose × fasting insulin)/22.5.

### Estimation of glycogen level

The glycogen content was measured using a commercial kit (Nanjing Jiancheng Bioengineering Institute, China). Briefly, hepatic tissues, muscle tissues, epididymal fat tissues, and subcutaneous fat tissues were homogenized in KOH solution (w/v, 30%) and dissolved for 20 min at 100 °C. Then, the detection reagent was added and glycogen was assessed following the manufacturer’s protocols. The value of glycogen level was shown as mg/g tissue.

### Measurement of G-6-Pase, PEPCK, and GS activities

The activities of G-6-Pase, GS, and PEPCK in hepatic tissue were quantified using the commercially available kits (Nanjing Jiancheng Bioengineering Institute, China) following the manufacturer’s protocols. The values were shown as IU/L.

### Serum lipid profile analysis

The levels of TG, TC, LDL-C, HDL-C, and free fatty acid (FFA) in serum were measured by corresponding assay kits (Nanjing Jiancheng Bioengineering Institute, China) following the manufacturer’s protocols.

### Quantitative real-time polymerase chain reaction (qPCR)

For qPCR analysis, total RNA was extracted from liver tissue using the Trizol reagent (Thermo Fisher Scientific, Waltham, MA, USA). Total RNA was retro-transcribed into cDNA by the ReverAid first-strand cDNA synthesis kit (Thermo Fisher Scientific, Waltham, MA, USA). Then, quantitative real-time PCR was performed using the SYBR Green reagent (Thermo Fisher, Waltham, MA, USA) in an ABI 7500 Thermal Cycler (Life Technologies, Waltham, MA). Primers were used for measuring gene expression in liver tissue shown in Table 1. The qPCR was carried out in duplicate, and the relative gene expression was quantified using the 2^−ΔΔCt^ method, and results were normalized using GAPDH.

**Table 1.** Primers for quantitative real-time PCR.

Gene	Forward primer	Reverse primer
Akt2	TCAGGATGTGGATCAGCGAGA	CTGCAGGCAGCGGATGATAA
PI3K	ATTGACAGTAGGAGGAGGTTGG	CTTTCTGCGTCAGCCACAT
GS	AGGACATTTCAGGGATTAA	GCCATCCATCTCCATCTGC
GLUT2	CATTGGCTGGAAGAAGCGTATCAG	GGAGACCTTCTGCTCAGTCGACG
G6PC	TTCACCTTCTCCTTCCCTGTAAGG	TACCAGCTTGAGCAGCACAAGTCG
PEPCK	AAGACTCCCAGGACTGGTTCAT	TAGCAGGTAGAATCCAAGCGCG
FOXO1	AGAGTTAGTGAGCAGGCTACAT	CCGCTGTTGCCAAGTCTGA
PPARα	GGAAACTGCCGACCTCAAAT	AACGAAGGGCGGGTTATTG
LDLR	CCAACCTGAAGAATGTGGTG	CAGGTCCTCACTGATGATGG
SREBP-1	CCCTGCGAAGTGCTCACAA	GCGTTTCTACCACTTCAGGTTTCA
ACC	ACACTGGCTGGCTGGACAG	CACACAACTCCCAACATGGTG
GAPDH	GAACGGGAAGCTCACTGGC	GCATGTCAGATCCACAACGG

### Western blot analysis

A RIPA lysis buffer was used to extract the total protein of liver tissue, and lysates were centrifuged at 10,000 × *g* for 15 min. The supernatant was collected for protein quantification by a bicinchoninic acid protein assay kit. Then, 40 µg of protein samples were separated by 5% SDS-polyacrylamide and transferred onto PVDF membrane. After that, the membranes were incubated with the following primary antibodies: anti-phospho-Akt; anti-Akt; anti-phospho-PI3K; anti-PI3K; anti-GLUT2 and anti-GAPDH (Cell Signalling Technology, Beverly, MA, USA; 1:1000 dilution) at 4 °C overnight. After washed with PBS, the membranes were incubated with appropriate secondary antibodies for another 2 h. The ImageJ software was used to quantify the blots.

### Subacute toxicity study of PLP in mice

Forty male mice were randomly separated into five groups (*n* = 8). The control group was administrated with saline daily by gavage for 14 consecutive days, while the experimental groups were treated with PLP daily at the doses of 312, 625, 1250, and 2500 mg/kg, respectively, for 14 consecutive days. At the end of treatment, all mice were sacrificed, and blood samples were collected and immediately centrifuged at 3000 *g* for 15 min to collect serum for the measurement of biochemical parameters.

### Data analysis

Data were expressed in the form of the means ± standard deviation (SD). GraphPad Prism software (GraphPad Software, Inc., La Jolla, CA, USA) was used for data analysis. Multiple comparisons were performed by one-way ANOVA followed by Tukey’s multiple comparison test. Differences were considered statistically significant if a *p* value of less than 0.05.

## Results

### Effects of PLP on body weight, tissue weight, and food intake in db/db mice

**Figure 1.**
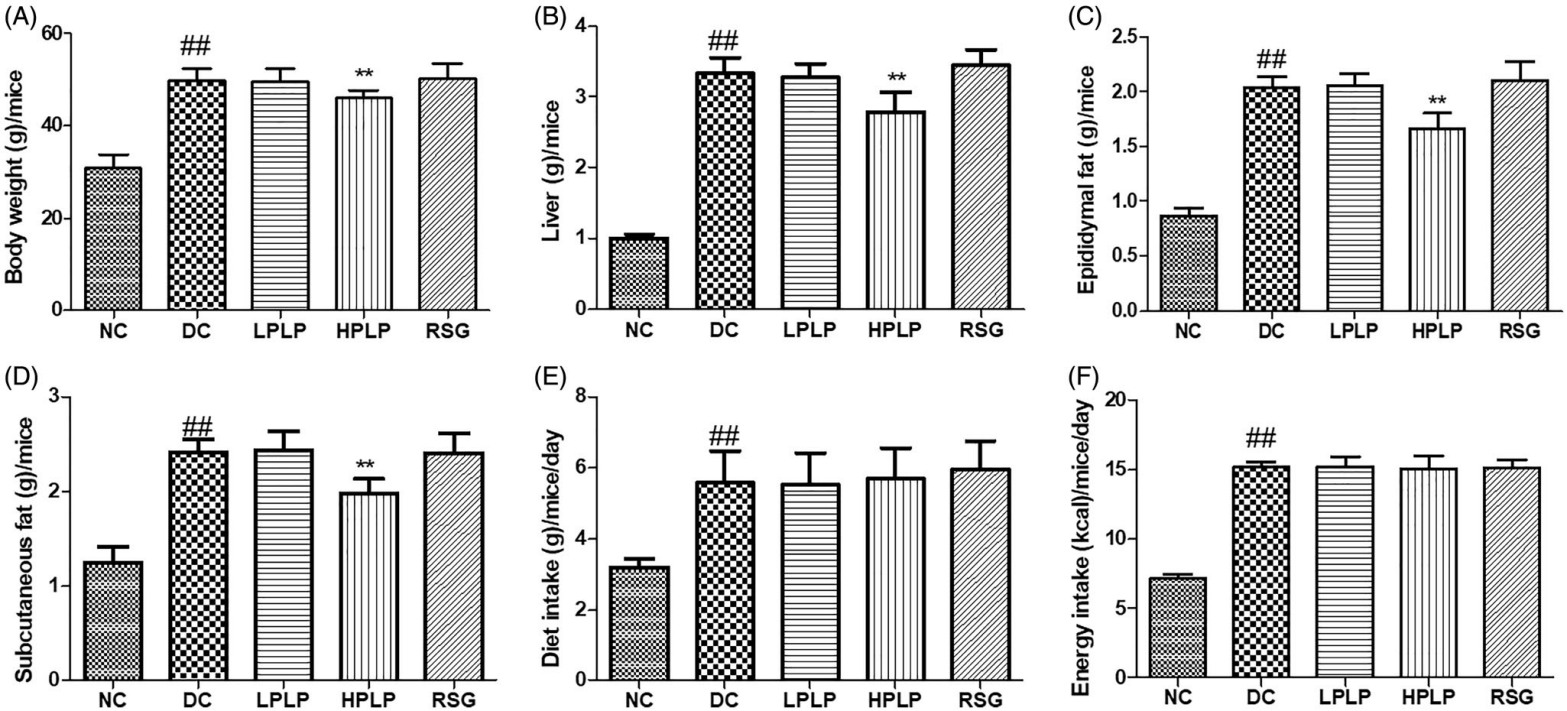
Effect of PLP for six weeks on: body weight (A), liver tissue (B), epididymal fat (C), subcutaneous fat (D), diet intake (E), and energy intake (F). Data are shown as the mean ± SD (*n* = 8). ^##^*p* < 0.01, DC group *vs.* NC group; **p < 0.01, drug-treated group *vs.* DC group.

As shown in Figure 1, the body weight, liver tissue, epididymal fat, and subcutaneous fat in the DC group were higher than those of the NC group (*p* < 0.01). PLP (100 mg/kg body weight) treatment decreased bodyweight, liver tissue, epididymal fat, and subcutaneous fat, compared to the DC group (*p* < 0.01). Compared with the NC group, the increases in diet intake and energy intake amount were found in the DC group (*p* < 0.01). However, there were no obvious differences between the DC group and the PLP group. The findings indicated that daily administration with PLP (100 mg/kg body weight) reduced body weight gain and fat accumulation without changing food and energy intake in db/db mice.

### PLP improved glucose and insulin tolerance in db/db mice

**Figure 2.**
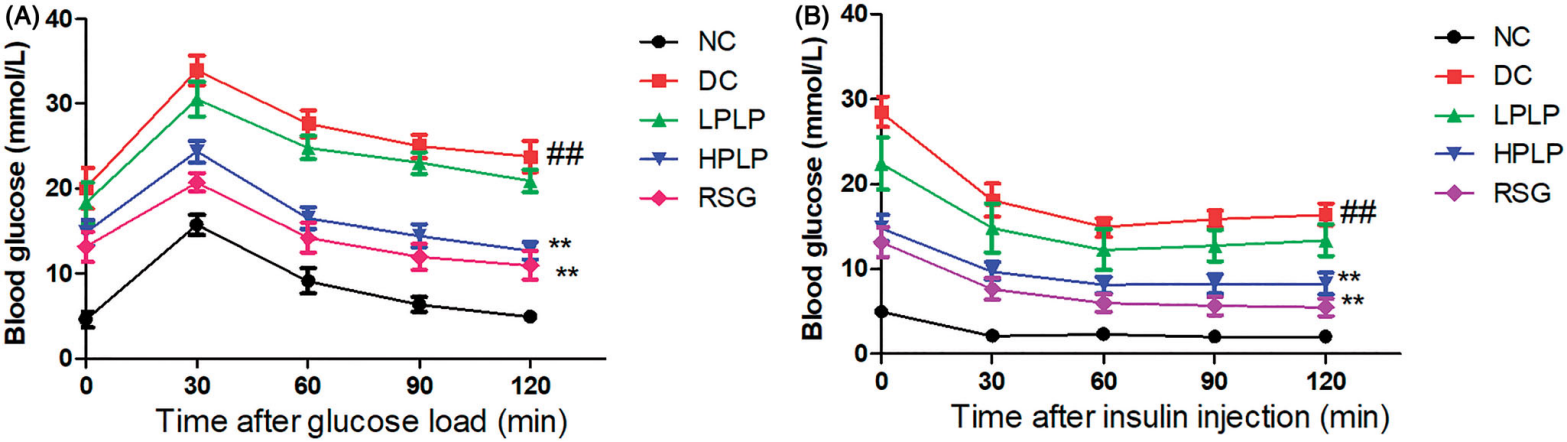
Effect of PLP treatment on the parameters of glucose tolerance and insulin resistance in db/db mice. Oral glucose tolerance test (OGTT) was carried out after six weeks of PLP treatment in all groups (A). Intraperitoneal insulin tolerance test (IPTT) was carried out after six weeks of PLP treatment in all groups. Data are shown as the mean ± SD (*n* = 8). ^##^*p* < 0.01, DC group *vs.* NC group; **p < 0.01, drug-treated group *vs.* DC group.

As shown in Figure 2(A), the blood glucose levels reached their peak value in all groups after oral administration of glucose for 30 min, and then the blood glucose levels decreased gradually within 120 min. Besides, the blood glucose levels in the DC group were always higher than that in the NC group throughout the OGTT test, while treatment with HPLP or RSG resulted in lower glucose levels than those observed in the DC group (30, 60, 90, and 120 min). Similarly, as shown in Figure 2(B), after injection of insulin for 60 min, the HPLP or RSG group exhibited lower blood glucose levels than those observed in the DC group (30, 60, 90, and 120 min). Therefore, our findings indicated that treatment of HPLP improved glucose and insulin tolerance in db/db mice.

### Effects of PLP on fasting blood glucose levels, insulin contents, and HOMA-IR in db/db mice

**Figure 3.**
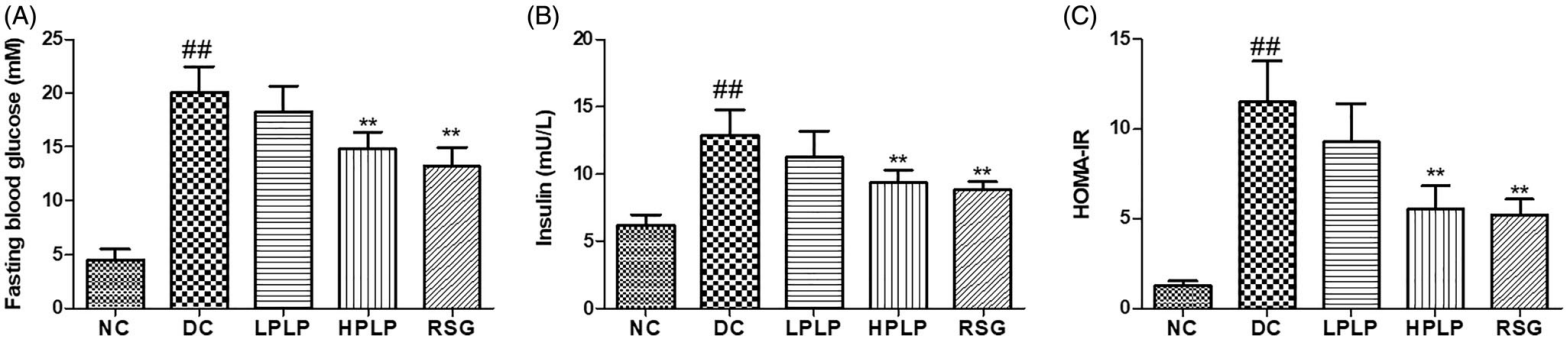
Effect of PLP on fasting blood glucose levels (A), insulin contents (B), and HOMA-IR (C) in db/db mice. Data are shown as the mean ± SD (*n* = 8). ^##^*p* < 0.01, DC group *vs.* NC group; **p < 0.01, drug-treated group *vs.* DC group.

As shown in Figure 3, compared with the NC group, serum fasting blood glucose levels and insulin contents in the DC group were increased (*p* < 0.01), indicating that insulin resistance was severe in the DC group. Administration with HPLP or RSG for six weeks, HPLP and RSG group mice exhibited declined serum glucose levels, insulin levels, and HOMA-IR index (*p* < 0.01), indicating that PLP (100 mg/kg body weight) could improve insulin resistance in db/db mice.

### PLP treatment alleviated insulin resistance via the regulation of enzyme activities and gene expression related to glucose metabolism

**Figure 4.**
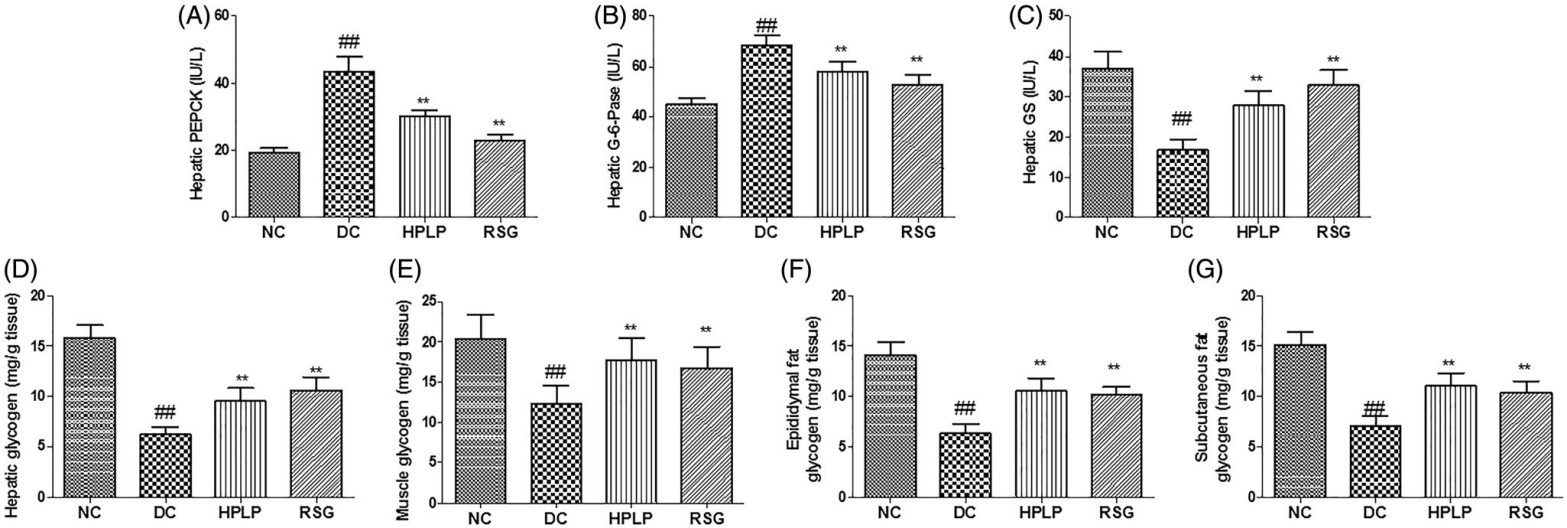
Effect of PLP on hepatic glucose metabolism in db/db mice. The hepatic PEPCK activity (A), hepatic G-6-Pase activity (B), hepatic GS activity (C), hepatic glycogen content (D), muscle glycogen content (E), epididymal fat glycogen content (F) and subcutaneous fat glycogen content (G). Data are shown as the mean ± SD (*n* = 8). ^##^*p* < 0.01, DC group *vs.* NC group; **p < 0.01, drug-treated group *vs.* DC group.

Because PLP improved glucose intolerance and insulin resistance in db/db mice, the activities of PEPCK, G-6-Pase, GS, and hepatic glycogen level were next assessed by ELISA. As shown in Figure 4, the hepatic PEPCK and G-6-Pase activities were increased in the DC group compared to the NC group, while hepatic, muscle, epididymal fat, subcutaneous fat glycogen content, and GS activity were decreased (*p* < 0.01). Administration with HPLP or RSG for six weeks, the RSG and HPLP (100 mg/kg body weight) groups exhibited decreased the hepatic PEPCK and G-6-Pase activities, increased the hepatic, muscle, epididymal fat, subcutaneous fat glycogen content, and GS activity, compared to the DC group (*p* < 0.01).

**Figure 5.**
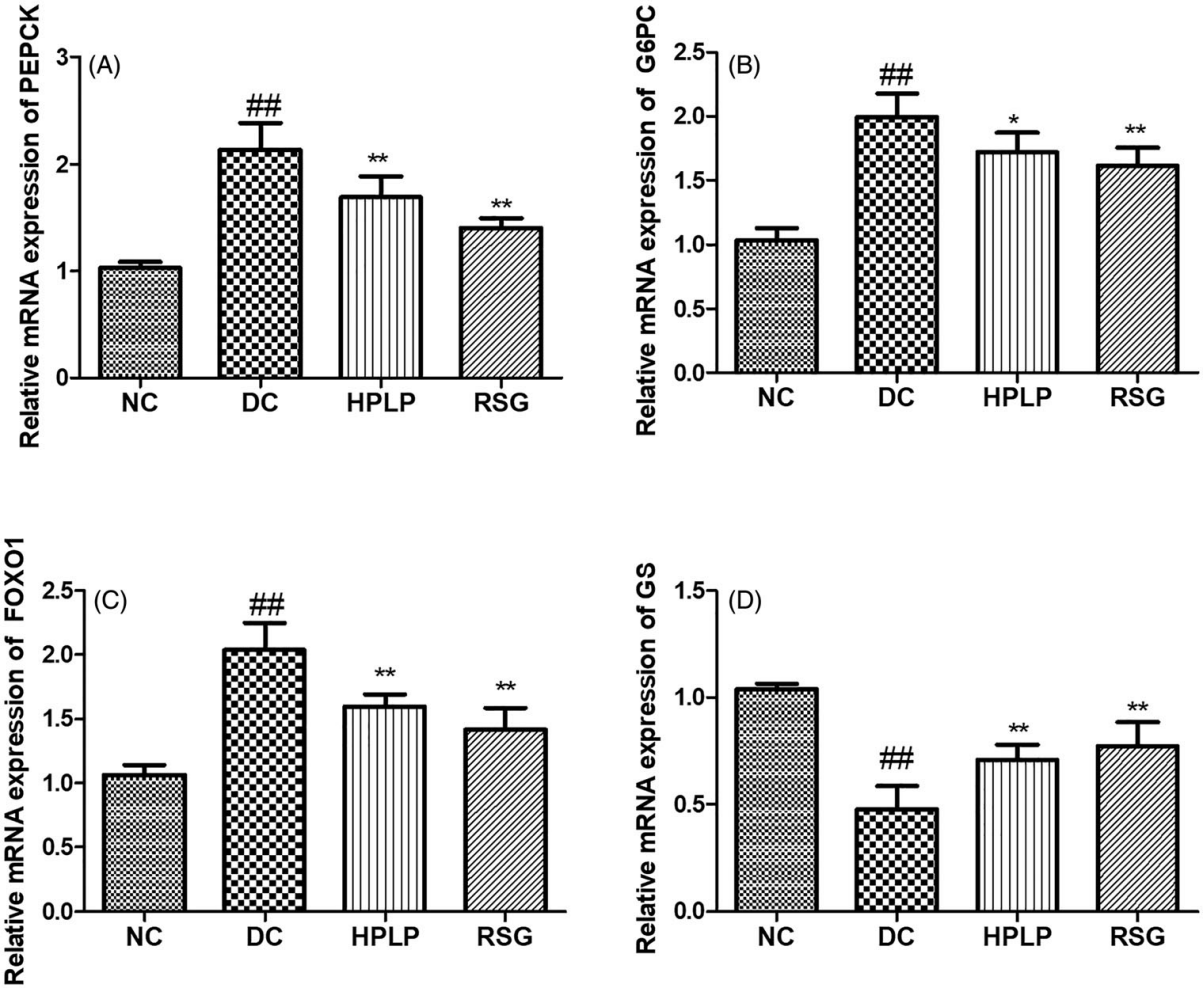
Effect of PLP on hepatic glucose metabolism-related gene expression levels in db/db mice. The relative mRNA levels of PEPCK (A), G6PC (B), FOXO1 (C), and GS (D) in the liver. Data are shown as the mean ± SD (*n* = 6). ^##^*p* < 0.01, DC group *vs.* NC group; **p < 0.01, drug-treated group *vs.* DC group.

The mRNA expression of PEPCK, G6PC, and GS was analysed to further verify the results of the PEPCK, G6PC, and GS activities (Figure 5). As expected, the mRNA expression levels of PEPCK, G-6-Pase, and FOXO1 were up-regulated in the DC group compared to the NC group, while the mRNA expression level of GS was down-regulated (*p* < 0.01). Administration with HPLP for six weeks, the mRNA expression levels of PEPCK, G-6-Pase, and FOXO1 were decreased, while the mRNA expression level of GS was increased, compared to the DC group (*p* < 0.01), indicating that PLP (100 mg/kg body weight) treatment inhibited gluconeogenesis and promoted glycogen synthesis of db/db mice liver tissue.

### PLP treatment activated the insulin signalling pathway in db/db mice

**Figure 6.**
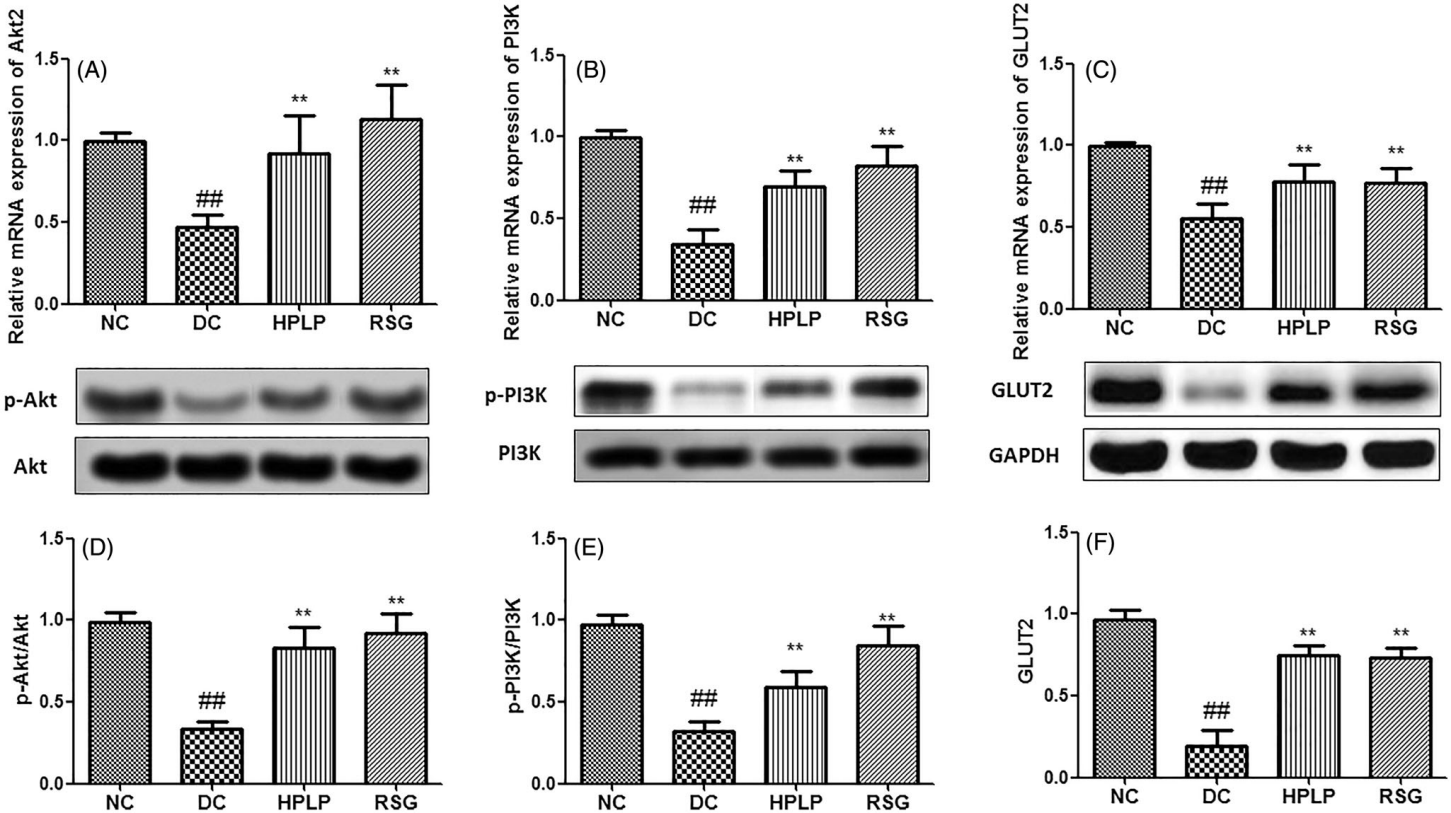
Effect of PLP on the Akt2/PI3K/GLUT2 insulin signalling pathway in db/db mice. The relative mRNA levels of Akt2 (A), PI3K (B), and GLUT2 (C) in the liver. The relative protein levels of p-Akt2 (D), p-PI3K (E), and GLUT2 (F) in the liver. Data are shown as the mean ± SD (*n* = 6). ^##^*p* < 0.01, DC group *vs.* NC group; **p < 0.01, drug-treated group *vs.* DC group.

As shown in Figure 6, the hepatic mRNA and protein expression of Akt2, PI3K, and GLUT2 in the DC group was declined when compared with the NC group (*p* < 0.01). Compared to the DC group, these expression levels were upregulated in the hepatic tissues after administration with HPLP for six weeks. These findings indicated that PLP alleviated insulin resistance in db/db mice possibly via the activation of the PI3K/AKT signalling pathway.

### Effects of PLP on serum lipid profiles in db/db mice

**Figure 7.**
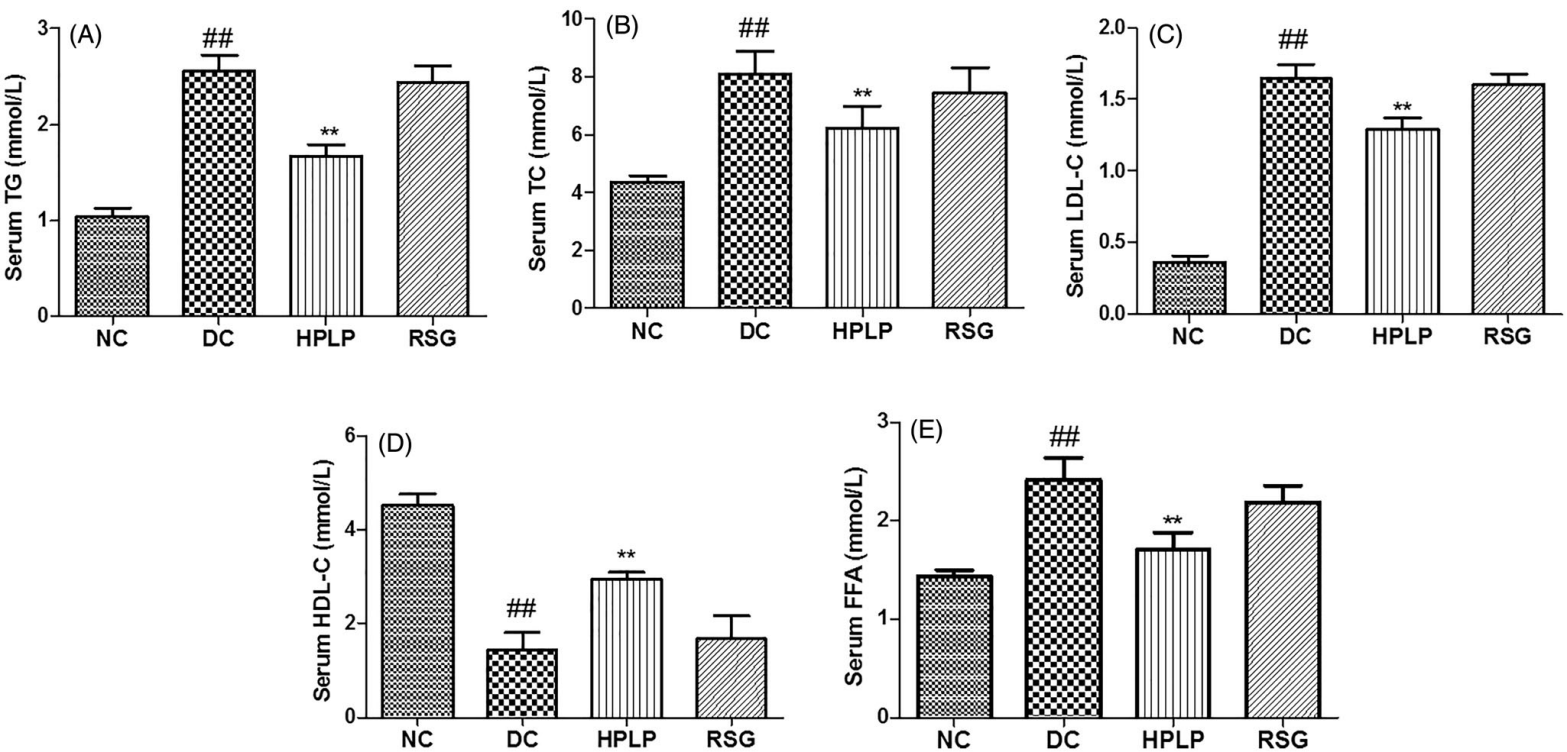
Effect of PLP on serum lipids profiles in db/db mice. The levels of TG (A), TC (B), LDL-C (C), HDL-C (D), and FFA (E) in the serum of mice. Data are shown as the mean ± SD (*n* = 6). ^##^*p* < 0.01, DC group *vs.* NC group; **p < 0.01, drug-treated group *vs.* DC group.

The serum TG, TC, LDL-C, HDL-C, and FFA levels were assayed in the present study to investigate the possible improvement effect of PLP on dyslipidemia in db/db mice. As shown in Figure 7, the serum HDL-C level was decreased in the DC group compared to the NC group, while TG, TC, LDL-C, and FFA levels were increased (*p* < 0.01). Administration with HPLP for six weeks, the RSG and HPLP (100 mg/kg body weight) groups exhibited increased the serum HDL-C level, decreased the serum TG, TC, LDL-C, and FFA levels, compared to the DC group (*p* < 0.01). These findings showed that PLP treatment could improve hyperlipidaemia in db/db mice.

### Effects PLP on hepatic lipid metabolism in db/db mice

**Figure 8.**
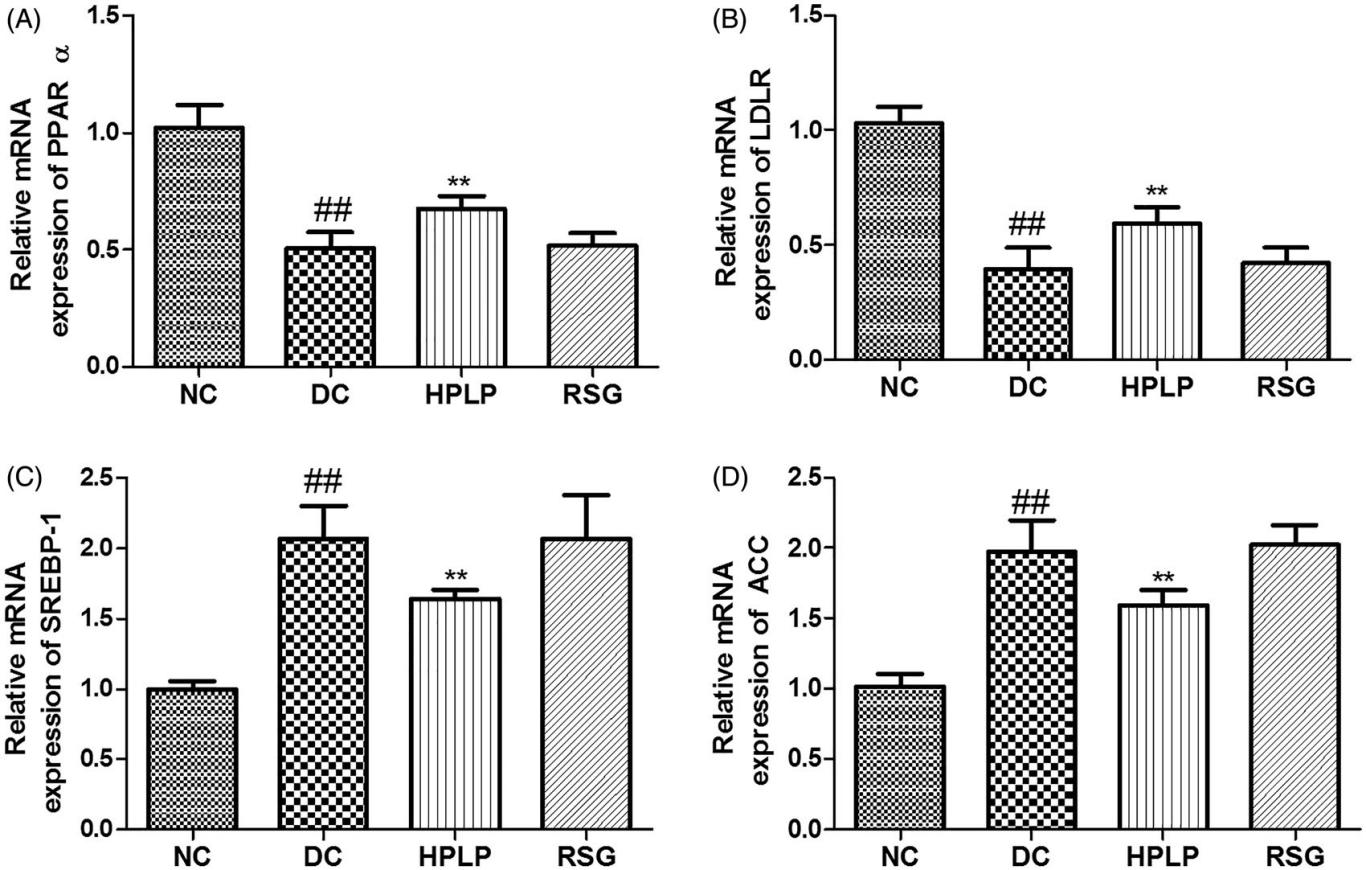
Effects PLP on hepatic lipid metabolism-related gene expression levels in db/db mice. The relative mRNA levels of PPARα (A), LDLR (B), SREBP-1 (C), and AAC (D) in the liver. Data are shown as the mean ± SD (*n* = 6). ^##^*p* < 0.01, DC group *vs.* NC group; **p < 0.01, drug-treated group *vs.* DC group.

As shown in Figure 8, the mRNA expression levels of PPARα and LDLR were down-regulated in the DC group compared to the NC group, while mRNA expression levels of SREBP-1 and ACC were up-regulated (*p* < 0.01). Administration with HPLP for six weeks, the mRNA expression levels of PPARα and LDLR were increased, while mRNA expression levels of SREBP-1 and ACC were decreased, compared to the DC group (*p* < 0.01), indicating that PLP (100 mg/kg body weight) treatment improved lipid metabolism in db/db mice.

### Subacute toxicity study

The results of the biochemical parameters following PLP administration for 14 consecutive days are shown in Table 2. There was no significant difference in the biochemical parameters (ALT, AST, BUN, CRE, TG, and TC) of mice between the control group and PLP treated groups (312, 625, and 1250 mg/kg). Therefore, mice were treated with PLP daily at the doses of 312, 625, and 1250 mg/kg bodyweight for 14 consecutive days are safe.

**Table 2.** Effects of PLP on serum biochemical results of mice during 14 consecutive days of subacute toxicity study.

Biochemical parameters	Control	PLP (312 mg/kg)	PLP (625 mg/kg)	PLP (1250 mg/kg)	PLP (2500 mg/kg)
ALT (U/L)	39.49 ± 3.11	39.99 ± 3.08	40.50 ± 3.06	41.58 ± 2.89	43.57 ± 2.90*
AST (U/L)	130.70 ± 11.68	132.60 ± 10.22	133.20 ± 10.16	134.00 ± 10.21	137.40 ± 9.37
BUN (mmol/L)	6.71 ± 0.38	6.79 ± 0.37	6.87 ± 0.37	6.90 ± 0.38	7.16 ± 0.36*
CRE (mg/dL)	35.95 ± 0.78	36.03 ± 0.92	36.46 ± 0.92	36.80 ± 0.85	37.02 ± 0.82*
TG (mmol/L)	0.95 ± 0.07	0.96 ± 0.06	0.95 ± 0.05	0.97 ± 0.07	1.02 ± 0.04
TC (mmol/L)	4.05 ± 0.13	4.04 ± 0.11	4.06 ± 0.12	4.09 ± 0.13	4.14 ± 0.12

Data are shown as the mean ± SD (*n* = 8). *p < 0.01, drug treated group vs. Control group.

ALT: alanine aminotransferase; AST: aspartate aminotransferase; BUN: blood urea nitrogen; CRE: creatinine; TG: triglyceride; TC: total cholesterol; PLP: *Pueraria lobata* root polysaccharide.

## Discussion

The ideal hypoglycaemic agent is that improves insulin resistance and glucose metabolism with fewer side effects. However, the long-term use of chemically synthesized anti-diabetic drugs for the treatment of type 2 diabetes mellitus often leads to the side effects, such as acute kidney injury and abdominal discomfort (Filippatos et al. 2014). Thus, developing novel therapies is of great significance. Recent studies have indicated that compounds derived from Traditional Chinese Medicines have exerted potential hypoglycaemic effects in the preclinical study (Yan et al. 2016). *Pueraria lobata* has widely been used in Traditional Chinese Medicine for the treatment of diabetes and cardiovascular diseases. However, there are lots of different bioactive constituents in *Pueraria lobata*. Polysaccharides are one of the major bioactive constituents in *Pueraria lobata*. Thus, it is of great interest to investigate the benefits effects of PLP in Type 2 diabetes mellitus. Since db/db mice are characterized by hyperlipidaemia, hyperinsulinemia, hyperglycaemia, and insulin resistance due to the mutation of leptin receptor (Belke and Severson 2012). Insulin plays a vital role in maintaining blood glucose balance (Zhang et al. 2018). Thus, db/db mice were widely used as an appropriate model for Type 2 diabetes mellitus. In our study, we found that PLP decreased the blood glucose levels in the DC group, and this hypoglycaemic effect was as stable as RSG. Moreover, the OGTT and IPTT results indicated that PLP treatment obviously alleviated glucose tolerance and insulin sensitivity. Additionally, after administration with PLP for six weeks, the insulin level and HOMA-IR index were declined in the DC group. These findings indicated that PLP could ameliorate insulin resistance, subsequently promoting glucose metabolism, and declining fasting blood glucose levels.

The liver is one of the most important sites to regulate carbohydrate metabolism and plays an important role in maintaining blood glucose homeostasis (Han et al. 2019). It has been reported that insulin resistance affected the activity of carbohydrate metabolism-related enzymes, thus decreased the capacity of hepatic tissue to synthesis glycogen, utilize and decompose glucose. Eventually, more glucose is secreted into the blood. Our findings indicated that PLP could increase hepatic glycogen content in db/db mice, which implied that PLP could regulate hepatic glucose homeostasis. PEPCK and glucose-6-phosphatase (G6Pase) are the main rate-limiting enzymes involved in the regulation of liver gluconeogenesis. Glycogen synthase (GS) is one of the main rate-limiting enzymes involved in the regulation of hepatic glycogen synthesis (Ros et al. 2010). Our results indicated PLP treatment could suppress the activities of PEPCK and G6Pase enzyme, and downregulate the mRNA expression levels of PEPCK, G6PC, and FOXO1 in db/db mice, thus suppressing hepatic gluconeogenesis. This is consistent with a previous report (Valenti et al. 2008), we found that MOMA-IR index, and PEPCK and G6PC mRNA levels were positively correlated with FOXO1 mRNA level. Moreover, our findings also showed that PLP treatment could upregulate the mRNA expression level of GS, thus promoting glycogenesis. The above findings showed that the activities of carbohydrate metabolism-related enzymes were modulated to notably decrease blood glucose by PLP.

It has been reported that the PI3K/Akt insulin signalling pathway is involved in glucose uptake and metabolism in the liver, and blocks glucose secretion from hepatocytes (Song et al. 2014). Glucose transporters (GLUTs) are a wide group of membrane proteins and play a vital role in regulating glucose metabolism in the adipose, skeletal muscle, and liver tissue (Chadt and Al-Hasani 2020). GLUT2 specifically exists in the liver, which is involved in the transfer of glucose between the blood and the liver. As expected, the mRNA expressions of Akt2, PI3K, and GLUT2 were up-regulated by PLP treatment in db/db mice. The findings indicated that PLP could improve insulin resistance via activation of the PI3K/Akt signalling pathway in db/db mice.

Obesity is closely associated with insulin resistance and hyperglycaemia (Hirosumi et al. 2002). In general, fat accumulation could increase the secretion of free fatty acids (FFA) into the blood in the condition of insulin resistance. Excessive FFA deposits in the liver, skeletal muscle, and pancreatic islet tissue in the form of TG leading to the impairment of tissue and further increasing insulin resistance (Xia et al. 2014). Our findings indicated that treatment with PLP decreased body weight gain and fat accumulation without changing food and energy intake in db/db mice. These findings corresponded well with a previous study that indicated that the oral administration of *Rosa roxburghii* polysaccharide could decrease the body weight gain in db/db mice (Zhang et al. 2018).

Hyperlipemia is strongly attributed to impairment of tissue and insulin resistance in type 2 diabetes mellitus. It has been reported that SREBP-1c plays an important role in TG accumulation and fatty acid biosynthesis via regulating the expression of acetyl-CoA carboxylase (ACC) (Batista et al. 2019). And activation of ACC resulted in the promotion of the synthesis of cholesterol and fatty acids which then inhibit fatty acid oxidation. Our results showed that the administration of PLP down-regulated the mRNA expression levels of SREBP-1c and ACC in db/db mice, indicating that the suppression of SREBP-1c and ACC contributed to the bodyweight loss via inhibiting lipid accumulation, which is consistent with the previous report (Ju et al. 2015). Besides, PPARα is a primary transcriptional regulator that regulates genes involved in fatty acid oxidation and lipid metabolism (Zandbergen and Plutzky 2007). LDLR plays an important role in effective lipid homeostasis, the upregulation of LDLR expression will cause the decline of plasma LDL-C levels (Petroglou et al. 2020). The gene expression levels of PPARα and LDLR were up-regulated in db/db mice after the treatment with PLP, implying that PLP could regulate live lipid homeostasis via promoting lipid oxidation and regulating plasma lipid profiles. In the present research, our findings indicated that PLP could improve glucose and lipid metabolism, and promotes weight loss in db/db mice, all of which contribute to ameliorate insulin resistance. In contrast, although RSG improved glucose metabolism in the present study, this drug is devoid of weight loss effects.

## Conclusions

We have provided evidence that PLP derived from *Pueraria lobata* root, could alleviate a series of metabolic disorders, including glucose intolerance, insulin resistance, hyperglycaemia, and dyslipidemia in db/db mice without body weight gain. These beneficial metabolic effects of PLP are partly related to the activation of the GLUT2/PI3K/Akt signalling pathways. These findings implied that PLP might potentially be used as an antidiabetic agent for the prevention and treatment of metabolic syndrome in type2 diabetes mellitus.

## Data Availability

The datasets used and/or analysed during the current study are available from the corresponding author on reasonable request.
